# Structural Analysis of Human and Mouse Dendritic Spines Reveals a Morphological Continuum and Differences across Ages and Species

**DOI:** 10.1523/ENEURO.0039-22.2022

**Published:** 2022-06-07

**Authors:** Netanel Ofer, Ruth Benavides-Piccione, Javier DeFelipe, Rafael Yuste

**Affiliations:** 1 Neurotechnology Center, Department Biological Sciences, Columbia University, New York, NY 10027; 2 Laboratorio Cajal de Circuitos Corticales, Centro de Tecnología Biomédica, Universidad Politécnica de Madrid, Madrid, 28223, Spain; 3 Instituto Cajal, Consejo Superior de Investigaciones Científicas, Madrid 28002, Spain

**Keywords:** 3D confocal reconstructions, cerebral cortex, plasticity, pyramidal cells

## Abstract

Dendritic spines have diverse morphologies, with a wide range of head and neck sizes, and these morphologic differences likely generate different functional properties. To explore how this morphologic diversity differs across species and ages we analyzed 3D confocal reconstructions of ∼8000 human spines and ∼1700 mouse spines, labeled by intracellular injections in fixed tissue. Using unsupervised algorithms, we computationally separated spine heads and necks and systematically measured morphologic features of spines in apical and basal dendrites from cortical pyramidal cells. Human spines had unimodal distributions of parameters, without any evidence of morphologic subtypes. Their spine necks were longer and thinner in apical than in basal spines, and spine head volumes of an 85-year-old individual were larger than those of a 40-year-old individual. Human spines had longer and thicker necks and larger head volumes than mouse spines. Our results indicate that human spines form part of a continuum, are larger and longer than those of mice, and become larger with increasing adult age. These morphologic differences in spines across species could generate functional differences in biochemical and electrical spine compartmentalization, or in synaptic properties, across species and ages.

## Visual Abstract

**Figure:**
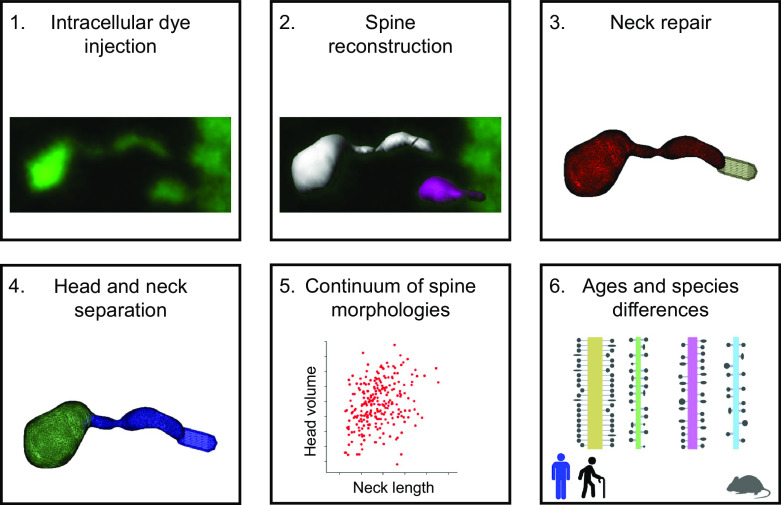

## Significance Statement

Dendritic spines mediate most excitatory contacts in the brain and enable biochemical and electrical compartmentalization. Spine morphologies are diverse and this diversity likely has functional consequences. Here, we use unsupervised algorithms to computationally dissect spine heads and necks and systematically measure morphologic features of spines from light microscopy (LM) datasets of mouse and human cortex. Human spines form part of a continuum, without evidence of subtypes. Human spines also have longer and thicker necks and bigger head volumes than mouse spines. Our results demonstrate a rich morphologic diversity of human spines, and systematic differences across ages and species.

## Introduction

Dendritic spines, first described by Cajal (Cajal, 1888, 1904) and are considered key elements in learning, memory, and cognition (Yuste, 2010, 2015). Spines are sites of most excitatory synapses in many brain areas, and practically all spines receive at least one excitatory synapse (Arellano et al., 2007; Harris and Weinberg, 2012; DeFelipe, 2015). Thus, spine numbers and shape likely influence cortical functions. Differences in spine density and size between cortical areas and species exist (Elston et al., 2001, 2011; Jacobs et al., 2001, 2002; Ballesteros-Yáñez et al., 2010). For example, spines in human temporal cortex have higher density, head size, and neck length than those of mice (Benavides-Piccione et al., 2002; Ballesteros-Yáñez et al., 2010). However, mouse prelimbic cortex has similar spine density as human (Ballesteros-Yáñez et al., 2010). In human neurons, spines on apical dendrites are denser, larger, and longer than those on basal dendrites (Benavides-Piccione et al., 2013). Also, age-dependent functional changes in human cortex could be mediated by age-related spine loss (Rakic et al., 1994; Jacobs et al., 1997; Hof and Morrison, 2004; Petanjek et al., 2008; Benavides-Piccione et al., 2013; Dickstein et al., 2013). Indeed, small, short spines from basal dendrites and long spines from apical dendrites are lost with age (Benavides-Piccione et al., 2013).

Light microscopy (LM) has been traditionally used to reconstruct spine structures, enabling the dynamic tracking of spine morphologies in living neurons (Fischer et al., 1998; Dunaevsky et al., 1999; Tønnesen et al., 2014; Loewenstein et al., 2015; Bokota et al., 2016; Dickstein et al., 2016; Kashiwagi et al., 2019), revealing that spines are dynamic, changing size and shape over timescales of seconds, because of actin-based motility (Fischer et al., 1998; Dunaevsky et al., 1999; Hering and Sheng, 2001). However, previous LM studies were based on manual measurements, as automatic detecting, segmenting, and measuring spines from LM images is challenging (Levet et al., 2020; Okabe, 2020). Fluorescence signals from spines are often weak, making it difficult to identify clear spine borders. Also, the spine neck is at times invisible because of its small length and diameter, often below the optical resolution limit. Thus, separating the head and neck is particularly challenging. Electron microscopy (EM) solves these issues, and enables high-resolution nanometer-scale 3D morphologic analysis of dendritic spines (Ofer et al., 2021) and the reconstruction of spines located directly above or below the dendritic shaft (Parajuli and Koike, 2021). But, despite recent advances, large-scale automated serial EM still requires significant resources and time, even to reconstruct small tissue volumes. In contrast, confocal microscopy allows the rapid visualization of thousands of spines with high signal-to-noise (Benavides-Piccione et al., 2013). Thus, automatic methods to measure spines structure in LM databases could enable the systematic analysis of spine morphologies in living tissue.

To develop an automatic analysis pipeline for LM images of spines, we applied unsupervised algorithms to computationally separate and measure spine heads and necks from LM images of a dataset of 3D reconstructed human and mouse dendritic spines. These tools build on previous spine computational repairing method (Luengo-Sanchez et al., 2018) and algorithms for spine reconstructions from EM data (Ofer et al., 2021). Using these computational methods, we systematically examined morphologic variables of apical and basal dendritic spines from pyramidal neurons from cingulate cortex of two human individuals of different ages, and compared those measurements with those of pyramidal neurons from mouse somatosensory cortex. Our results reveal a continuum of spine morphologies, without any clear subtypes, and confirm significant differences in spine structures between species, with human spines being systematically longer and larger. These morphologic differences imply the existence functional differences in synaptic and spine function across species.

## Materials and Methods

### Human spines dataset

**Figure 1.**
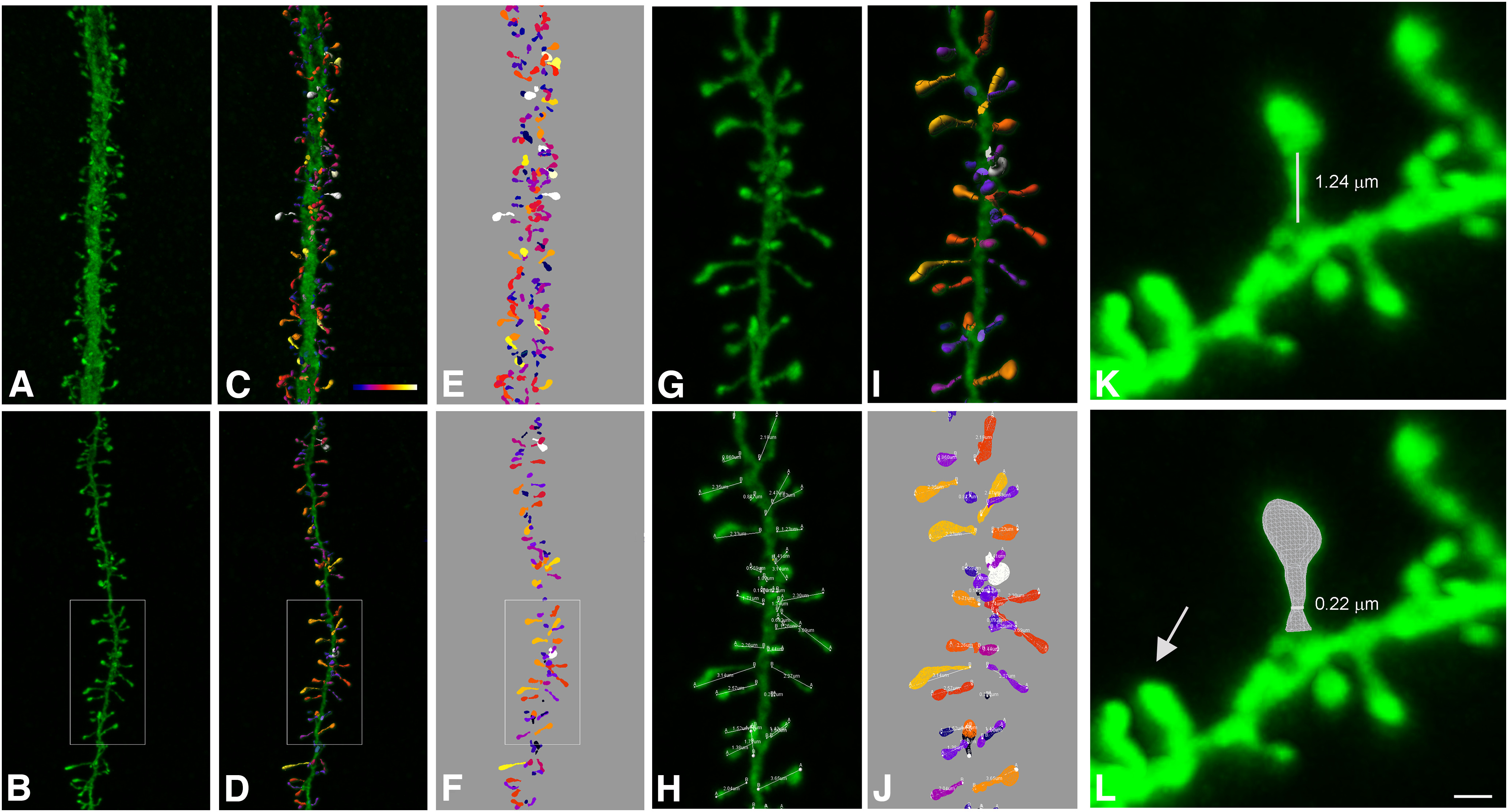
Spine reconstruction from confocal microscopy images. ***A***, ***B***, Apical (***A***) and basal (***B***) dendritic segments from intracellularly injected layer 3 pyramidal neurons from the human cingulate cortex. ***C***, ***D***, 3D reconstruction of each dendritic spine from the dendritic segment shown in ***A***, ***B***. Estimation of the spine volume values is shown by color codes (blue-white: 0–0.896 μm^3^). ***E***, ***F***, Surface meshes that were manually created for each individual spine. ***G–J***, Higher magnification images of the dendritic segment shown in ***B***. Spine lengths (that were measured in 3D) are also illustrated in ***H***, ***J***. ***K***, Example of a typical spine showing a clear head and neck. White line shows neck length. ***L***, Illustrates the corresponding surface mesh. White line shows neck diameter. Arrow indicates a spine not showing a clear head and neck. Dynamic scale bar (presented in ***L***): 5 μm (***A–F***), 2 μm (***G–J***), and 0.6 μm (***K***, ***L***).

We used a database of 7917 3D-reconstructed spines (Fig. 1; published previously; Benavides-Piccione et al., 2013), from intracellularly injected apical and basal dendrites from layer three pyramidal neurons in the cingulate cortex of two human males aged 40 and 85 obtained at autopsy. Briefly, brains were immersed (2–3 h postmortem), in cold 4% paraformaldehyde in 0.1 m phosphate buffer, pH 7.4 (PB) and sectioned into 1.5-cm-thick coronal slices. Vibratome sections (250 μm) from the anterior cingular gyri (corresponding to Brodmann’s area 24; see Zilles and Amunts, 2010) were obtained with a vibratome and labeled with 4′,6′-diamino-2-phenylindole (DAPI; Sigma) to identify cell bodies. Pyramidal cells were then individually injected with Lucifer yellow (LY; 8% in 0.1 m Tris buffer, pH 7.4), and thereafter immunostained for LY using rabbit antisera against LY (1:400,000; generated at the Cajal Institute). Apical and basal dendrites were imaged at high magnification (63× glycerol; voxel size: 0.075 × 0.075 × 0.28 μm^3^) using tile scan mode in a Leica TCS 4D confocal scanning laser attached to a Leitz DMIRB fluorescence microscope. Voxel size was calculated to acquire images at the highest resolution possible for the microscope (∼200 nm). Consecutive image stacks (approximately three) were acquired to capture the full dendritic depth, length, and width of basal dendrites, each originating from a different pyramidal neuron (10 per case; 60 dendritic segments). For apical dendrites, the main apical dendrite was scanned, at a distance of 100 μm from the soma up to 200 μm (8 dendrites per case; 16 dendritic segments). Apical and basal spines were individually reconstructed in 3D from high-resolution confocal stacks of images, using Imaris (Bitplane AG), by selecting a solid surface that matched the contour of each dendritic spine. Sometimes it was necessary to use several surfaces of different intensity thresholds to capture the complete morphology of a dendritic spine, resulting in fragmented spines. Spine length was also measured, using the same software, as manual measurement from the point of insertion within the dendritic shaft to the end of the spine (see Fig. 1*H*). Further information regarding tissue preparation, injection methodology, immunohistochemistry, imaging, 3D reconstruction, and ethics statement details is outlined previously (Benavides-Piccione et al., 2013).

### Mouse spines dataset

Mouse tissue samples were obtained from C57BL/6 adult (eight-week-old) male mice (*n* = 4). Animals were overdosed by intraperitoneal injection of sodium pentobarbitone and perfused via the heart with PBS (0.1 m) followed by 4% paraformaldehyde in PB. Brains were then removed and further immersed in 4% paraformaldehyde for 24 h. Coronal sections (200 μm) were obtained with a vibratome, which included the hindlimb somatosensory cortical region (S1HL; Gould et al., 2012). Layer three pyramidal cells were then intracellularly injected and immunostained as specified above. Thereafter, apical and basal dendrites were also scanned (63× glycerol; voxel size: 0.075 × 0.075 × 0.14 μm^3^; three apical and seven basal dendritic segments) and a total of 1683 spines 3D reconstructed as described above.

### Experimental design and statistical analysis

#### Spine reconstruction

The 3D mesh of each dendritic segment with all their spines was converted from the specific Imaris format (VRML file) into OBJ format files using Neuronize2 (Velasco et al., 2020). The default parameters were used: output resolution percentage: 30.0, precision: 50, union level: 3, include segments. The reconstruct process includes joining all the pieces into one solid mesh per spine, rasterization, dilation, erosion, and a reconstruction from a binary image by 3D meshes voxelization (Eyal et al., 2016). No image preprocessing was performed on the confocal data. To correct the z-distortion caused by confocal stacks, the z-dimension values were multiplied by a factor of 0.84 (Benavides-Piccione et al., 2013).

#### Head and neck separation

For each triangle of the mesh, we calculated two local parameters: the shape diameter function (SDF) and the distance from the triangle face to the closest point along the mesh skeleton, as described previously (Ofer et al., 2021). A Gaussian mixture model was used to classify the triangles into head or neck. Specifically, the cluster with the lower average SDF value was labeled as “neck” and the other cluster was labeled as “head.” These factors, combined with a spatial factor that takes into account the dihedral angle between neighboring faces, were applied in an energy-function graph-cut-based algorithm. Segmentation between head and neck was implemented using the Computational Geometry Algorithms Library (CGAL) 5.0.2, https://www.cgal.org; Triangulated Surface Mesh Segmentation package. Since spines reconstructed from LM images were smoother than those reconstructed from EM, we chose the smoothing-λ parameter to be 0.5.

**Figure 2.**
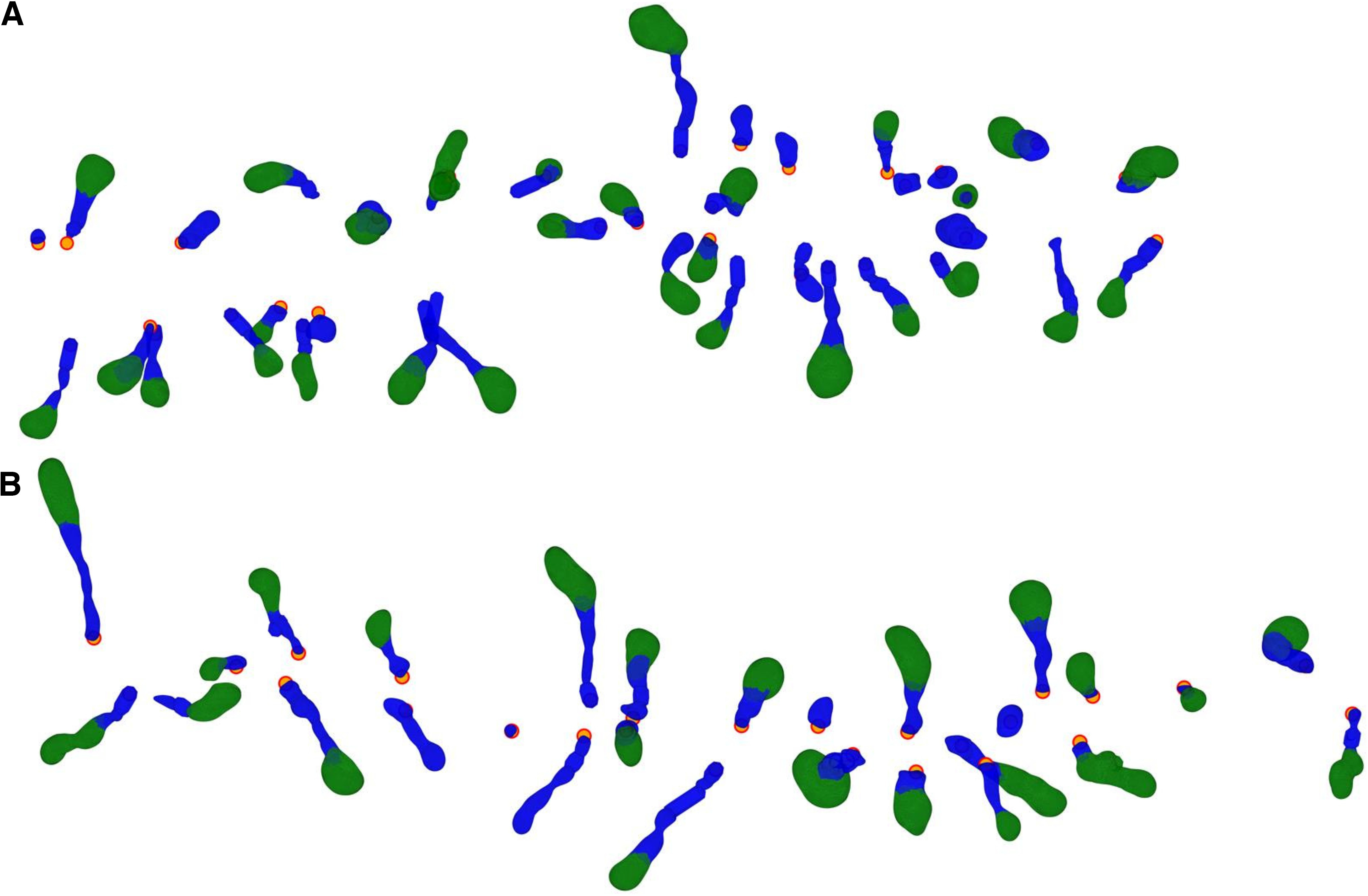
Computational separation of spine heads and necks. ***A***, Human apical spines, the same dendritic shaft that is presented in Figure 1*A*. ***B***, Human basal spines, the same dendritic shaft that is presented in Figure 1*B*. Spine heads in green and spine necks in blue. The orange dots indicate the insertion point of the spine into the dendritic shaft.

**Figure 3.**
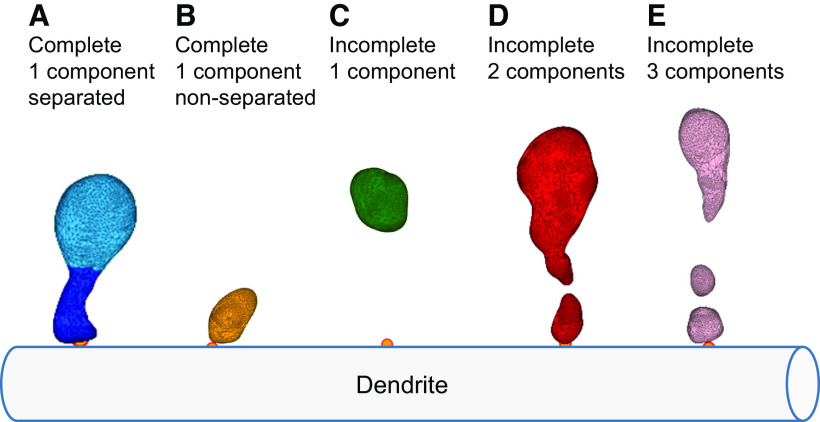
Spine dataset classification. ***A***, Complete spines, consisting of a single component that can be separated into the head and neck. ***B***, Complete spines, consisting of a single component that could not be separated into the head and neck. ***C***, Incomplete one component spines, detached from the dendritic shaft, containing mainly the head. ***D***, Incomplete spines consisting of two components. ***E***, Incomplete spines consisting of three components. The orange points indicate spine insertion on the dendritic shaft.

The morphologic parameters of the head and neck were measured as described previously (Ofer et al., 2021). The spine head sphericity was calculated by Equation 1:

(1) $$Sphericity=\frac{\pi^{\frac{1}{3}}{\left(6V\right)}^{\frac{2}{3}}}{A},$$

where *V* is the spine head volume and *A* is the area. Examples of spines separated into head and neck are presented in Figure 2. This separation algorithm enables the distinction between spines that have head and neck (Fig. 3, group A) and spines that do not clearly have head and neck (Fig. 3, group B).

#### Spine neck repair

A challenging process in spine morphologic reconstruction is the neck, which may not be visible because of its small diameter, under the resolution limit of LM. In case where it could not be detected we computationally repaired the neck. We defined five groups of spine reconstructions: (A) complete spines attached to the dendritic shaft with a single component that can be separated into head and neck (Fig. 3*A*); (B) complete spines attached to the dendritic shaft, with a single component that cannot be separated into head and neck (Fig. 3*B*); (C) incomplete spines with one component, representing mainly the spine head, not attached to the dendritic shaft (Fig. 3*C*); (D) incomplete spines with two disconnected components (Fig. 3*D*); and (E) incomplete spines with three or more disconnected components (Fig. 3*E*).

First, we analyzed only the complete spines with clear head and neck separation (group A), representing 20% of the mouse spines and 44% of the human spines in our dataset. To further increase the number of spines in the analysis, we computationally repaired the incomplete spines (groups C and D). Adding repaired spines resulted in 60% of the spines that could be analyzed in mice and in humans. Spine meshes that contained three or more components (Fig. 3*E*, group E) were discarded from the analysis, because of their complex shape and their small weight in the data (4% of mouse spines and 1% of human spines).

**Figure 4.**
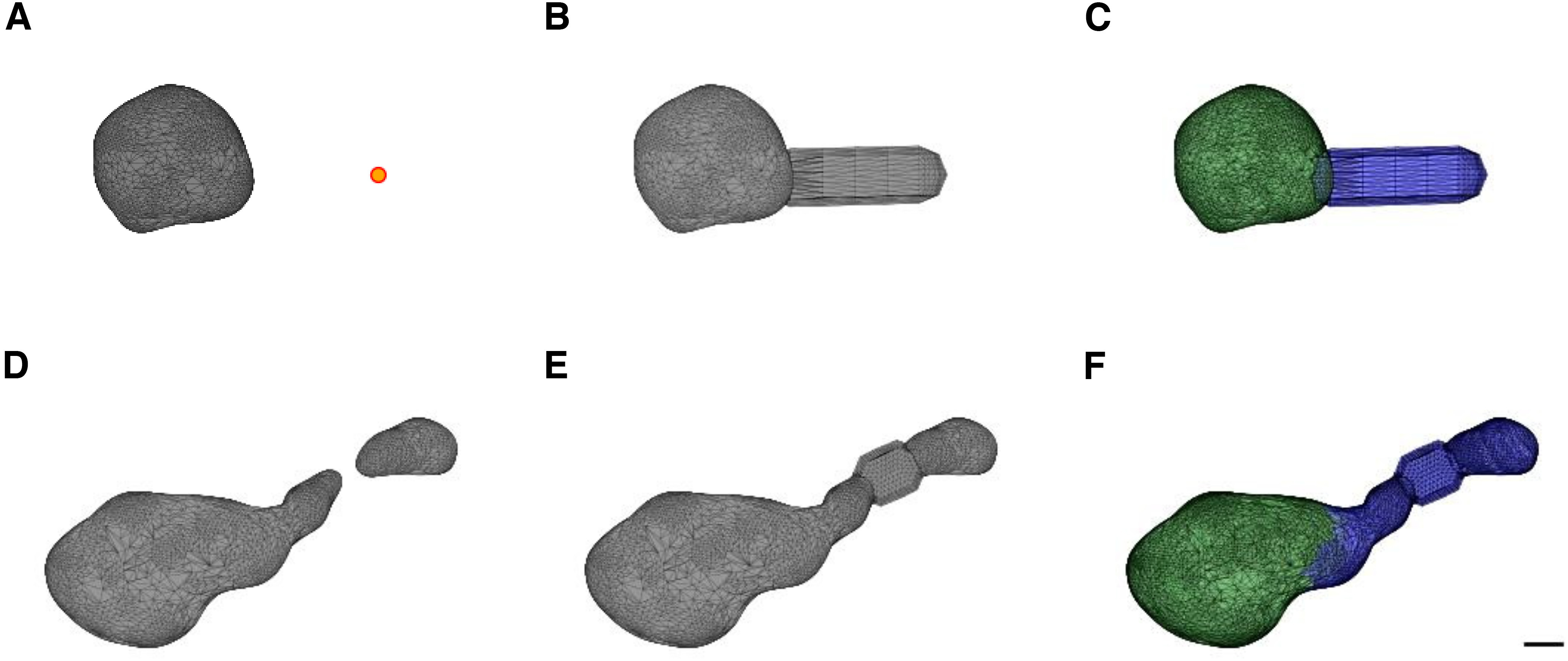
Spine neck repair of group C and group D spines. ***A***, Incomplete spine mesh from group C. The orange dot indicates the base point, between the spine neck and the dendritic shaft. ***B***, Repair of the neck by adding a cylinder between the base point and the closest vertex of the head. This cylinder is not the real neck; it was added just to enable measuring the neck length and separating the head and neck. ***C***, Separation into head (green) and neck (blue). ***D***, Incomplete spine mesh, consisting of two components (group D). ***E***, Repair of the neck by adding a cylinder between the two components, resulted in a connected spine. ***F***, Separation into head (green) and neck (blue). Scale bar: 200 nm. Extended Data Figure 4-1 shows the comparison between the morphologic parameters of only the complete spines (group A) and the complete and repaired spines (groups A, C, and D).

Fragmented or detached spines were reconstructed in a previous work by applying a closing morphologic operator or by applying a 2D Gaussian filter (Luengo-Sanchez et al., 2018). However, since here we were interested in the separation of the spine head and neck, we developed another approach. To repair incomplete spines where the neck is invisible (Figs. 3*C*, 4*A*, group C), we used the spine insertion points on the dendritic shaft (Fig. 4*A*, orange point). When the anchor point was far away, at least 200 nm from the closest vertex of the mesh (in spines belonging to group C and not to B), we repaired the neck by adding a simple cylinder between them (Fig. 4*B*). This enabled us to measure the neck length and allowed the separation into the head and neck. To repair disconnected spines (Figs. 3*D*, 4*D*, group D, meshes that contain two components) we patched the two closest points of the separated meshes by a simple cylinder (Fig. 4*E*), re-connecting the mesh into a single component. The radius of the cylinder used was the median neck radius measured from spines separated into head and neck before the repair process (group A), 140 nm for mice and 170 nm for humans. Although this constant radius is likely not accurate for all spines, it enabled us to segregate the head and neck, without affecting the head volume (Extended Data Fig. 4-1). From repaired spines (Fig. 4*C*,*F*, groups C and D), we measured only head volume and neck length, but not the neck diameter. Analysis of complete spines (group A) is presented in the main text of the paper, whereas analysis of both complete and repaired spines (groups A, C, and D) is presented in the Extended Data material.

10.1523/ENEURO.0039-22.2022.f4-1 Extended Data Figure 4-1 The repair process extends necks without changing head sizes. Head volume and neck length distributions of the complete spines (group A, continuous lines) and including the repaired spines (groups A, C, and D, dashed lines). ***A***, ***B***, Basal spines of the 40-year-old individual, *n* = 1359, 1012. ***C***, ***D***, Apical spines of the 40-year-old individual, *n* = 925, 430. ***E***, ***F***, Basal spines of the 85-year-old individual, *n* = 949, 670. ***G***, ***H***, Apical spines of the 85-year-old individual, *n* = 623, 424. Vertical lines indicate the median values. Mann–Whitney *U* rank test, the asterisks indicate statistical significance; ***p < 0.001. Download Figure 4-1, EPS file.

Adding a neck (to repair group C spines) was conducted for 28% of the mouse spines and 19% of the human spines, whereas re-connection of the two-component spine (to repair group D spines) was performed in 18% of mouse spines and 12% of human spines. In some spines (5.6% in mouse spines and 3.4% in human spines) both procedures were used, a connection of the two separated components followed by a further elongation of the neck. After the repair process the human and mouse dataset contained 7044 (89%) and 1536 (91%) spines, respectively.

#### Statistical analysis

**Figure 5.**
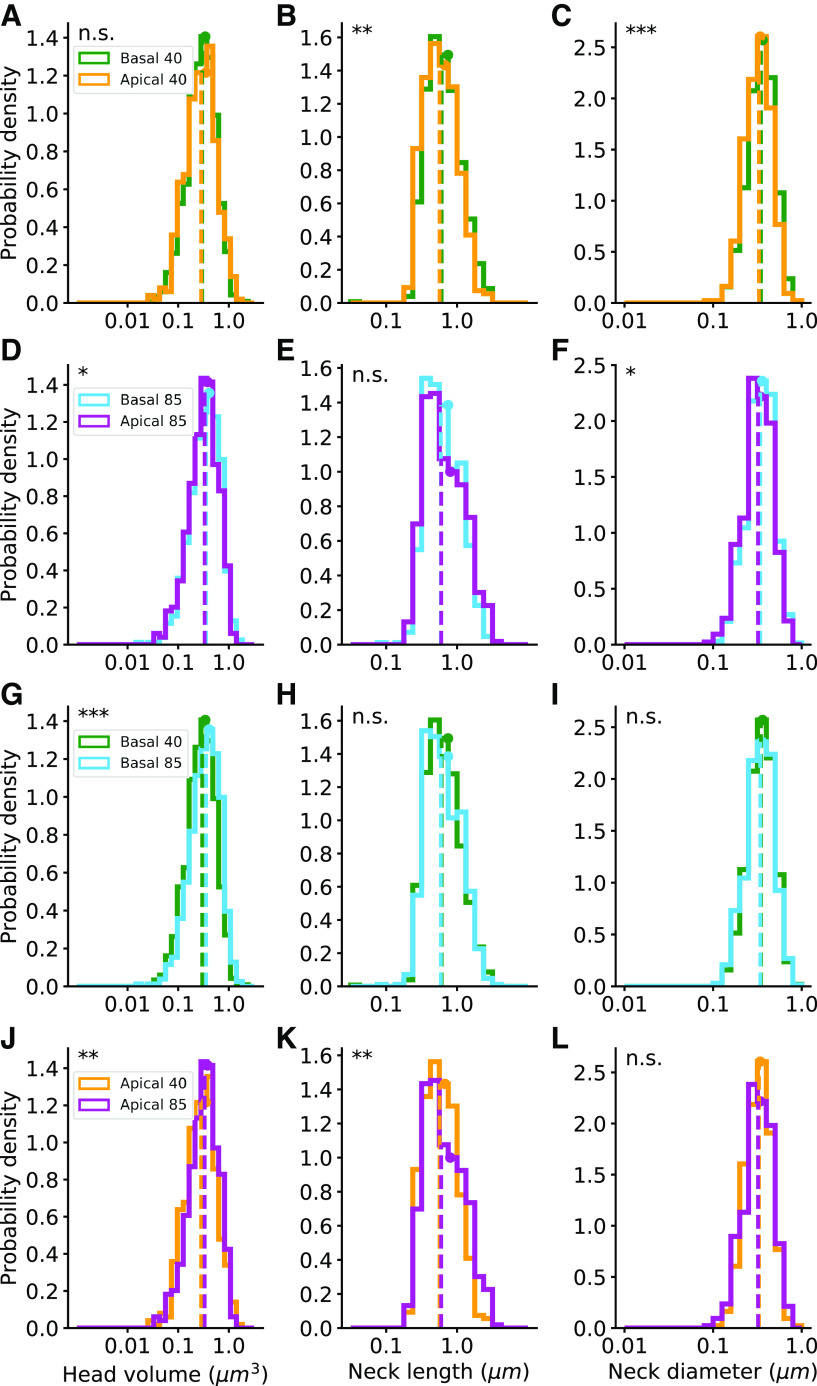
Human spines: morphologic parameter distributions. The distributions of the head volumes, neck lengths, and neck diameters of complete spines (group A). ***A–C***, Comparison between basal and apical spines of the 40-year-old-individual. ***D–F***, Comparison between basal and apical spines of the 85-year-old individual. ***G–I***, Comparison between 40- and 85-year-old basal spines. ***J–L***, Comparison between 40- and 85-year-old apical spines. Dashed vertical lines indicate the median values and the circles indicate the average. Apical 40 *n* = 430, basal 40 *n* = 1012, apical 85 *n* = 424, and basal 85 *n* = 670. Mann–Whitney *U* rank test, the asterisks indicate statistical significance; *p < 0.05, **p < 0.01, ***p < 0.001. Extended Data Figure 5-1 shows the distributions of the head volumes and neck lengths for the complete and repaired spines (groups A, C, and D). Extended Data Figure 5-2 shows the intraindividual analysis of the dendrites from the 40- and the 85-year-old humans.

**Figure 6.**
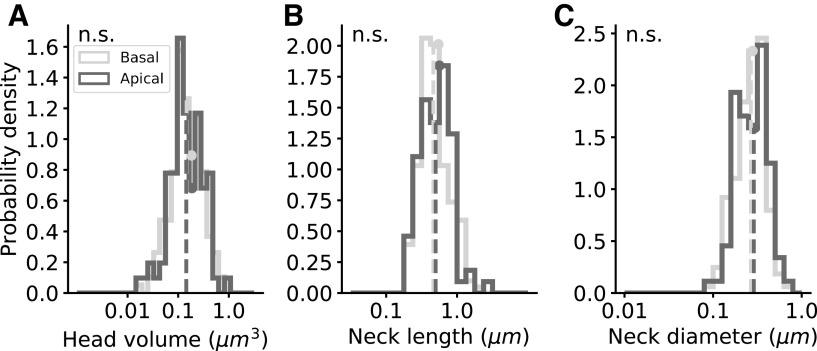
Mouse spines: morphologic parameter distributions. The distributions of complete spines (group A). ***A–C***, Head volume, neck length, and neck diameter distributions of basal (light gray, *n* = 163) and apical (dark gray, *n* = 88) spines. Dashed vertical lines indicate the median values and the circles indicate the average. *p* = 0.48, 0.23, 0.2; Mann–Whitney *U* rank test. Extended Data Figure 6-1 shows the results for the complete and repaired spines (groups A, C, and D).

**Figure 7.**
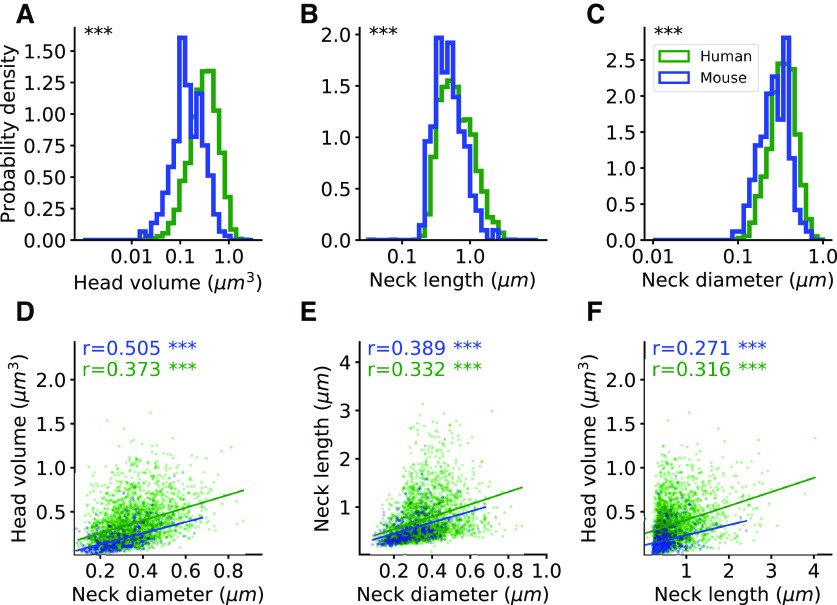
Spine morphologic distributions of humans and mice. ***A–C***, Head volume, neck length, and neck diameter distributions in human (green, *n* = 2536) and mouse (blue, *n* = 251) complete spines (group ***A***). Mann–Whitney *U* rank test, ***p < 0.001. ***D–F***, Correlation between spine head and neck morphologic variables. The correlation coefficients (Spearman) are indicated for each graph. The asterisks indicate statistical significance; **p < 0.01, ***p < 0.001. Two-sided *p*-value for a hypothesis test whose null hypothesis is that the slope is zero, using Wald test with *t* distribution of the test statistic. Extended Data Figure 7-1 shows the results for the complete and repaired spines (groups A, C, and D). Extended Data Figure 7-2 shows the correlation between head volume and neck length separately for different ages and dendrites.

Mann–Whitney *U* rank test was used to compare groups and to calculate differences between two groups with quantitative values. A nested ANOVA was performed followed by Tukey’s HSD test to explore potential differences between spines of the two different-age individuals and across dendrites of each individual. The *y*-axes of the histograms (Figs. 5-7) represent the probability density, where each bin displays the bin’s raw count divided by the total number of counts and the bin width, so that the area under the histogram integrates to 1. This representation allows the comparison between different-size groups. Because of the skewed distribution of the data, the histograms are plotted in a logarithmic scale, to allow detection of potential bi-modalities and comparison between different populations. Linear scale histograms are presented in Extended Data Figure 7-1*A–C*. Hartigan’s dip test was used to test the unimodality of the data (Hartigan and Hartigan, 1985). The correlation between parameters was examined by the Wald test with *t* distribution of the test statistic. The two-sided *p*-value for a hypothesis test whose null hypothesis is that the slope is zero. The asterisks indicate statistical significance: n.s, not significant, *p < 0.05, **p < 0.01, ***p < 0.001.

10.1523/ENEURO.0039-22.2022.f5-1 Extended Data Figure 5-1 Human spines morphological parameter distributions in the complete and repaired spines (groups A, C, and D). The distributions of the head volumes and neck lengths. ***A***, ***B***, Comparison between basal and apical spines of the 40-year-old individual. ***C***, ***D***, Comparison between basal and apical spines of the 85-year-old individual. ***E***, ***F***, Comparison between 40- and 85-year-old spines in the basal spines. ***G***, ***H***, Comparison between 40- and 85-year-old spines in the apical spines. Dashed lines indicate the median values and the circles indicate the average. Apical 40 *n* = 925, basal 40 *n* = 1,359, apical 85 *n* = 623, and basal 85 *n* = 949. Mann–Whitney *U* rank test, the asterisks indicate statistical significance; **p < 0.01, ***p < 0.001. Download Figure 5-1, EPS file.

10.1523/ENEURO.0039-22.2022.f5-2 Extended Data Figure 5-2 Intraindividual analysis of the dendrites from the 40- and 85-year-old humans. Tukey’s pairwise multiple comparison test for the head volume, neck length, and neck diameter in apical and basal dendrites; complete spines (group A). Red squares indicate that the two varieties are significantly different (p < 0.05). Download Figure 5-2, TIF file.

### Code accessibility

The CGAL scripts were written in C++; the other codes were written in Python 3.7 using the libraries numpy 1.17.4, scipy 1.5.4, trimesh 3.9.8, and statsmodels 0.11.1. Nested ANOVA analysis was performed using the aov()function in R. Codes used are publicly available at our GitHub page (https://github.com/NTCColumbia/Spines-Morphologies-Confocal).

## Results

### Human spines have different morphologies depending on dendritic tree and age

We first compared spine head volume, neck length, and neck diameter on apical and basal dendrites from human samples. We found similar distributions of head volumes in basal and apical spines from the 40-year-old individual (Fig. 5*A*, *p* = 0.39), and a small but significant larger head volumes in basal spines of the 85-year-old individual (median of 0.356 μm^3^ compared with 0.333 μm^3^; Fig. 5*D*, *p* = 0.04). The neck lengths in the basal spines from 40-year-old individual were longer than those from apical spines (median of 0.607 μm compared with 0.566 μm; Fig. 5*B*, *p* < 0.01). In the 85-year-old individual, the median neck lengths of basal and apical spines had similar distributions (0.591 μm and 0.598 μm, respectively; Fig. 5*E*, *p* = 0.3). However, basal spines neck diameters were thicker than those of apical spines in samples from both the 40-year-old individual (median of 346 and 329 nm, respectively; Fig. 5*C*, *p* < 0.001) and the 85-year-old individual (median of 341 and 321 nm, respectively; Fig. 5*F*, *p* < 0.05).

Comparing the spines from the two individuals revealed statistically significant differences (Fig. 5*G–L*). Basal spines of the 85-year-old individual had bigger head volumes than those from the 40-year-old individual (median of 0.356 μm^3^ compared with 0.298 μm^3^; Fig. 5*G*, *p* < 0.001). Also, the 85-year-old apical spine heads were larger than those of the 40-year-old (median of 0.333 μm^3^ compared with 0.285 μm^3^; Fig. 5*J*, *p* < 0.01). The neck length in basal dendrites had a similar distribution in both individuals (median of 0.591 and 0.607 μm in the 85- and 40-year-old individuals, respectively; Fig. 5*H*, *p* = 0.38), whereas in the apical spines of the 85-year-old individual, longer necks were observed (median of 0.598 μm compared with 0.566 μm; Fig. 5*K*, *p* < 0.01). The spine neck diameters of the 85- and 40-year-old individuals were similar in basal (median of 341 and 346 nm, respectively; Fig. 5*I*, *p* = 0.2) and in apical (median of 321 and 329 nm, respectively; Fig. 5*L*, *p* = 0.49).

Given the fact that all samples came from only two individuals, we extended the statistical analysis by dividing datasets from each individual into datasets of different dendrites (8 apical and 30 basal dendritic segments for each individual). Consistent with the original datasets, nested ANOVA analysis also showed significant differences between ages in apical neck length (*p* < 0.001) and basal head volume (*p* < 0.001). No significant differences were found in apical head volume (*p* = 0.07) and neck diameter (*p* = 0.99), and basal neck length (*p* = 0.93) and neck diameter (*p* = 0.4). Tukey’s pairwise multiple comparisons between the subgroups showed statistical differences in spine morphology between the dendrites of the two individuals (Extended Data Fig. 5-2).

All the described above parameters of the complete spines (group A), including the average, standard deviation, median, and range are summarized in Table 1. The head volume and neck length values from all the spines, including also the repaired spines (groups A, C, and D), are presented in Extended Data Figure 5-1. The head volumes medians including the repaired spines were 0.298 μm^3^ in basal and 0.297 μm^3^ in apical spines of the 40-year-old individual, and 0.354 μm^3^ in basal and 0.341 μm^3^ in apical spines of the 85-year-old individual. The neck lengths medians including the repaired spines were 0.738 μm in basal and 0.884 μm in apical spines of the 40-year-old individual, and 0.734 μm in basal and 0.787 μm in apical spines of the 85-year-old individual (Extended Data Table 1-1). The ranges of the parameter distributions were similar in basal and apical dendrite and for both ages.

**Table 1:** Morphologic parameters values of human complete (group A) apical and basal spines from the 40- and 85-year-old individuals

	Age		Average ± STD	Median	Range
Head volume (μm^3^)	40	Apical	0.3526 ± 0.25	0.2846	0.0274–1.6268
		Basal	0.3426 ± 0.22	0.2978	0.0324–2.206
	85	Apical	0.3816 ± 0.23	0.3331	0.0378–1.3154
		Basal	0.4103 ± 0.25	0.3559	0.0189–1.538
Neck length (μm)	40	Apical	0.6739 ± 0.4	0.5664	0.1839–3.1336
		Basal	0.7464 ± 0.46	0.6074	0.0353–2.9899
	85	Apical	0.8025 ± 0.56	0.5976	0.1865–3.7792
		Basal	0.7483 ± 0.47	0.5911	0.0818–4.0137
Neck diameter (nm)	40	Apical	339.9 ± 114.36	328.7	97.4–836.4
		Basal	361.67 ± 122	346.32	113.5–868.4
	85	Apical	340.08 ± 120	320.98	99.7–732.6
		Basal	356.66 ± 124	340.62	100.9–811.8

Since neck diameters below the resolution limit (∼200 nm) cannot be visualized using LM, these values correspond to those spines that were completely visualized (60%). Extended Data Table 1-1 shows the morphologic parameters values for the complete and repaired spines (groups A, C, and D).

10.1523/ENEURO.0039-22.2022.tab1-1 Extended Data Table 1-1 **The head volume and neck length values of the apical and basal spines from the 40- and 85-year-old individuals of the complete and repaired spines (groups A, C, and D)** Download Table 1-1, DOCX file.

### Mouse spines show similar morphologies in apical and basal dendrites

We also compared the basal and apical complete spines (group A) in mice, finding similar distributions of the head volumes, neck lengths, and neck diameters (Table 2). Head volumes medians were 0.146 μm^3^ in basal and 0.144 μm^3^ in apical spines (Fig. 6*A*, *p* = 0.48), neck lengths medians were 0.463 μm in basal and 0.499 μm in apical spines (Fig. 6*B*, *p* = 0.23), and neck diameter medians were 267.1 nm in basal and 288.1 nm in apical spines (Fig. 6*C*, *p* = 0.2). All spines, including complete and repaired spines (groups A, C, and D), showed similar distributions of the head volumes and neck lengths of apical and basal dendrites (Extended Data Fig. 6-1; Extended Data Table 2-1). Head volumes medians including repaired spines were 0.124 μm^3^ in basal and 0.138 μm^3^ in apical spines, and the neck lengths medians were 0.587 μm in basal and 0.638 μm in apical. In conclusion, in mouse samples we did not find differences in spine head and neck dimensions between basal and apical dendrites, in contrast to the human data.

**Table 2:** Morphologic parameters of apical (*n* = 88) and basal (*n* = 163) spines from complete mouse spines (group A)

		Average ± STD	Median	Range
Head volume (μm^3^)	Apical	0.1875 ± 0.14	0.1441	0.0151–0.9041
	Basal	0.1832 ± 0.13	0.1468	0.0161–0.7097
Neck length (μm)	Apical	0.5627 ± 0.33	0.4994	0.0195–2.4059
	Basal	0.5467 ± 0.31	0.463	0.2074–2.2687
Neck diameter (nm)	Apical	295.86 ± 116	288.06	92.41–640.53
	Basal	278.55 ± 102	267.1	88.61–676.45

Since neck diameters below the resolution limit (∼200 nm) cannot be visualized using LM, these values correspond to those spines that were completely visualized (60%). Extended Data Table 2-1 shows the morphologic parameters values for the complete and repaired spines (groups A, C, and D).

10.1523/ENEURO.0039-22.2022.tab2-1 Extended Data Table 2-1 The **head volume and neck length values of the apical (*n *= 153) and basal (*n* = 290) spines from mice of the complete and repaired spines (groups A, C, and D)** Download Table 2-1, DOCX file.

10.1523/ENEURO.0039-22.2022.f6-1 Extended Data Figure 6-1 Mouse spines morphological parameter distributions in complete and repaired spines (groups A, C, and D). A, B, Head volume and neck length distributions of basal (light gray, n = 290) and apical (dark gray, n = 153) dendritic spines. The parameters were calculated only for the complete spines (group A). Dashed lines indicate the median values and the circles indicate the average; p = 0.17, 0.23; Mann–Whitney U rank test. Download Figure 6-1, EPS file.

### A continuum of spine morphologies in human and mouse spines

To explore whether human spines belonged to different morphologic subtypes, we analyzed the distribution of morphologic features. Each of the morphologic parameters showed skewed unimodal distributions (Fig. 7*A–C*; Extended Data Fig. 7-1*A–C*), with no clear signs of bimodality or multimodality. To test whether spines could be classified into different morphologic subtypes, we used Hartigan’s dip test on the head volume, neck length, and neck diameter (*p* = 1, 0.99, 0.99). Then, for each pair of parameters, we explored potential multimodal distributions (Fig. 7*D–F*), by constructing the 2D Hartigan’s dip-test that projects the scatter plot into histograms in several angles for head volume versus neck length (*p* = 0.83), head volume versus neck diameter (*p* = 0.92), and neck length versus neck diameter (*p* = 0.73). Further, using the 3D Hartigan’s dip-test (*p* = 0.93), we found a continuous and unimodal distribution. The lack of multimodality was also observed when examining each population separately (basal or apical, 40- or 85-year-old individuals; not shown). Similar results, were found in the mouse dataset, with a continuum of the morphologic parameters (Hartigan’s dip-test *p* = 0.74, 0.91, 0.97; 2D Hartigan’s dip-test *p* = 0.71, 0.44, 0.21; 3D Hartigan’s dip-test *p* = 0.72), consistent with previous EM results (Ofer et al., 2021).

10.1523/ENEURO.0039-22.2022.f7-1 Extended Data Figure 7-1 Comparing spine morphological distributions of humans and mice. A–C, Head volume, neck length, and neck diameter distributions in human (green, *n* = 2536) and mouse (blue, *n* = 251) complete spines (group A) in a linear scale. ***D***, ***E***, Head volume and neck length distributions in human (green, *n* = 3856) and mouse (blue, *n* = 443) complete and repaired spines (groups A, C, and D). ***F***, Correlation between spine head volume and neck length. The correlation coefficients (Spearman) are indicated. Two-sided *p*-value for a hypothesis test whose null hypothesis is that the slope is zero, using Wald test with *t* distribution of the test statistic. ***G***, ***H***, Spine volume and spine length distributions in humans (*n* = 3856, green) and mice (*n* = 443, blue). The thin dashed lines indicate the distribution of the all spines (groups A, B, C, and D), including those that are unseparated into head and neck, in humans (*n* = 7044) and mice (*n* = 1536). ***I***, The sphericity of the spine head in humans and mice (*p* = 0.077). Mann–Whitney *U* rank test, the asterisks indicate statistical significance; ***p < 0.001. Download Figure 7-1, EPS file.

10.1523/ENEURO.0039-22.2022.f7-2 Extended Data Figure 7-2 Spine morphological distributions of different age and dendrites of humans. Correlation between spine head volume and neck length of the complete and repaired spines (groups A, C, and D). ***A***, 40-year-old apical dendrite. ***B***, 85-year-old apical dendrite. ***C***, 40-year-old basal dendrite. ***D***, 85-year-old basal dendrite. The correlation coefficients (Spearman) are indicated. The asterisks indicate statistical significance; ***p < 0.001. Two-sided *p*-value for a hypothesis test whose null hypothesis is that the slope is zero, using Wald test with *t* distribution of the test statistic. Download Figure 7-2, EPS file.

The lack of multimodality in the morphologic parameters is not consistent with the existence of distinct morphologic spine types. We concluded that human cortical spines, like mouse ones, displayed a continuum distribution of morphologies.

### Human spines are larger and longer than those of mice

To explore species differences, we then compared human and mouse complete spines (group A); (Fig. 7*A–C*). Human spines ranged from 0.019–2.205 μm^3^ in head volume, 0.035–4.014 μm in neck length, and 97.4–868 nm in neck diameter. Meanwhile, mouse spines ranged from 0.015–0.904 μm^3^ in head volume, 0.02–2.406 μm in neck length, and 89–676 nm in neck diameter. Head volumes medians were 2.19 times larger in humans than in mice (0.32 vs 0.146 μm^3^), neck lengths medians were 1.27 times longer in humans (0.594 vs 0.469 μm), and neck diameters medians were 1.27 times wider in humans (339 vs 267 nm). When including repaired spines (groups A, C, and D), we also found bigger head volumes and longer necks in humans (Extended Data Fig. 7-1*D*,*E*). We also examined the distributions of the entire spine volume and length in humans and mice. For this purpose, we also included the spines that could not be separated into head and neck (group B) with the spines that could be separated into head and neck (groups A, C, and D; Extended Data Fig. 7-1*G*,*H*). The distributions of the sphericity of the spine head were the same in mice and humans, with an average of 0.86 (Extended Data Fig. 7-1*I*). Our results demonstrate that human spines have significantly larger head volumes and longer and thicker necks than mouse ones.

Finally, we explored the existence of potential correlations between spine morphologic parameters in human and mouse datasets. We found a significant positive correlation in the complete spines (group A) between head volume, neck length, and neck diameter in humans and mice (Fig. 7*D–F*). When including also the repaired spines (groups A, C, and D), we found no correlation between head volume and neck length in mice, and a weak correlation in humans (Extended Data Fig. 7-1*F*). The correlations between head volume and neck length in humans were observed for each age and dendritic compartment separately (Extended Data Fig. 7-2).

## Discussion

We used a computational pipeline to systematically analyze a confocal database of spines morphologies from human and mice cortical samples. These analyses supersede previous efforts, using mostly manual measurements, and enabled us to use a large dataset with close to 10 000 spines to quantitatively explore potential differences in spines morphologies between apical and basal dendrites, between mice and humans, and across humans of different ages. In human samples, we find that apical spines had longer and thinner necks than basal spines, but both populations had a similar distribution of head volumes. However, no differences were observed in spine head and neck dimensions between basal and apical dendrites in mouse pyramidal neurons. Interestingly, we found that spine head volumes from older human individuals were larger, both in apical and basal compartments. There was also a significant correlation between head volume, neck length, and neck diameter in humans and mice. All morphologic distributions of spine parameters, in both human and mouse samples, were unimodal, without any evidence for different spine morphologic subtypes. Finally, we showed that spine head volumes, neck lengths, and neck diameters are larger in humans than in mice.

### Methodological considerations

In many samples, it was difficult to detect spine necks. Indeed, ∼40% of humans or mice spines could not be clearly separated into head and neck (group B). This could be because of the fact that some spines do not have necks (stubby spines), perhaps because of the relatively young age of the mice (eight weeks; Helm et al., 2021). But a more likely possibility is the optical blurring of head and neck morphologies, because of the resolution limit of the LM. Indeed, super-resolution stimulated emission depletion (STED) microscopy in young mice (two to five weeks old), shows that spines that appear stubby in LM, are in fact short-necked spines (Tønnesen et al., 2014). Consistent with this, spine morphologic analyses with EM report <1% of spines without a clear neck (Parajuli et al., 2020; Ofer et al., 2021). Also in agreement with this, when examining spine volumes and lengths in all spines, including nonseparated spines (group B), we find a continuum distribution (Extended Data Fig. 7-1*G*,*H*, thin dashed curve), without statistical evidence for a subpopulation of spines without necks.

Capturing the morphologic features of the spine neck diameter, where the diameter is below the optical resolution limit, is impossible using LM. Thus, some spines (groups C and D) were not included in our data for neck diameter analysis. However, in the completed reconstructed spines (group A), neck diameters could be measured (Figs. 5, 6; Tables 1, 2). But, although the neck diameter measurements do not include the entire spine population, and within the limitations of the optical measurements of the spine neck, we can still gain insights by exploring this large dataset of spines.

Then, we developed a morphologic repair pipeline, to increase the analyzed head volume and neck length data (from 20% in mice and 44% in humans to around 60% in both). The repaired spines include spine neck length information from the thinnest spines (necks that tend to be invisible in LM). Importantly, the head volume does not differ when including the repaired spines, in contrast to neck length, as one would expect (Extended Data Fig. 4-1). Indeed, we found that in the repaired spines the neck lengths of the apical spines were longer than those of the basal spines, in contrast to the analysis of only the complete spines.

Comparing spine morphologic parameters obtained from different microscopy methods is difficult because of methodological differences. The spine dimensions we found are somewhat different from previously reported values, especially those that used super-resolution microscopy and EM (Tønnesen et al., 2014; Levet et al., 2020). We overall measured larger head volumes, shorter neck lengths, and thicker neck diameters. These differences are expected from the lower resolution of LM, whereby a spine head may be harder for the algorithm to isolate and could include part of the neck, leading to neck shortening. Also, the tortuous structure of the spine necks may not be captured by LM meshes, and thus, be interpreted as shorter. Moreover, the blurry border of the spine confocal image could be also interpreted as an enlarged spine. However, even with these limitations, the comparisons between spine populations and between samples that we perform in this study are still valid because we used the same methodology. Table 1 details a subset of the total human population of spines sampled, which include spines showing a clear differentiation between the head and neck, as well as spines in which this differentiation is not clear (Fig. 2). Therefore, these values cannot be directly compared with those of other studies, where spines display a clear spine head, showing longer and thinner necks (Eyal et al., 2018).

### Differences in morphologies in human basal and apical spines

**Figure 8.**
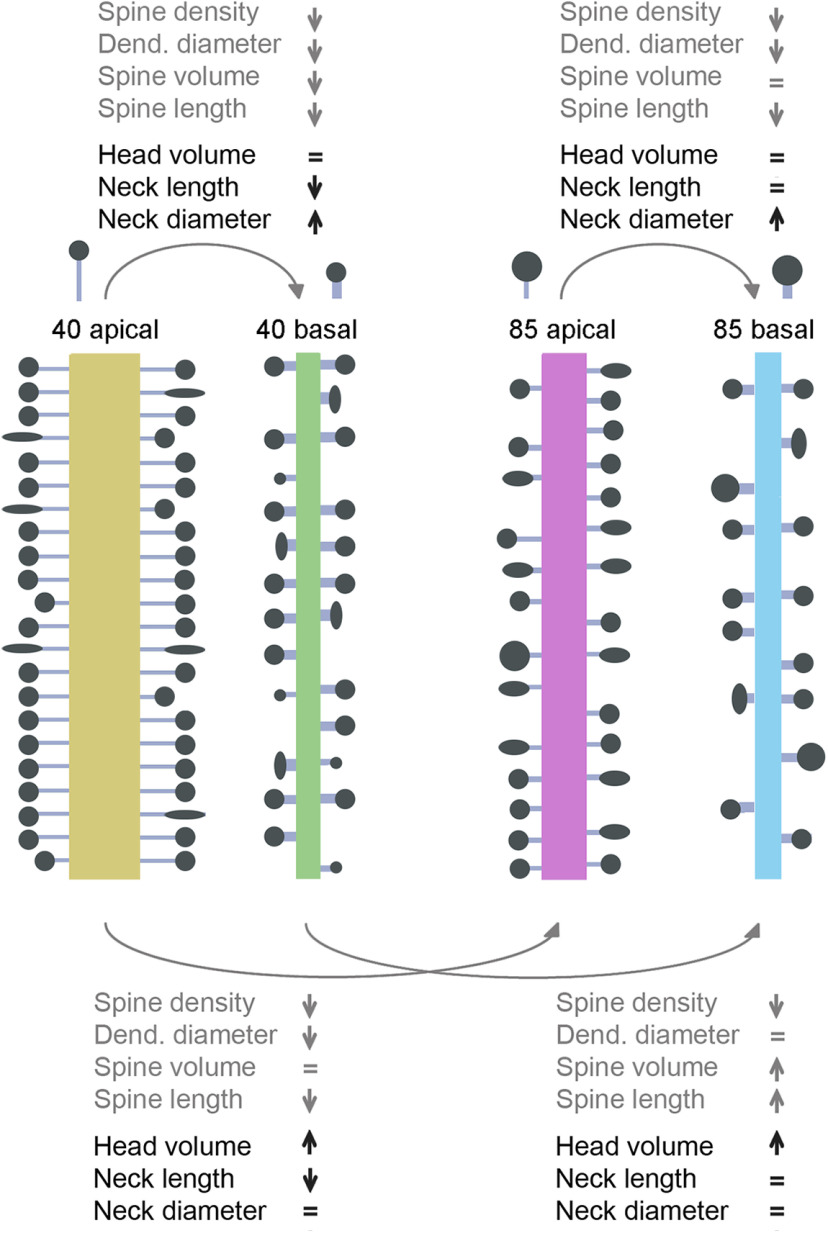
Differences in morphologic parameters, including complete and repaired spines (groups A, C, and D). Apical and basal dendritic compartments of the 40- and the 85-year-old human individuals. Dendritic shaft and spine properties from previous work (gray font; Benavides-Piccione et al., 2013) and spine properties from the present work (black font).

In our previous work, we found differences in spines morphologies between apical and basal dendrites in human spines. Specifically, apical dendrites had higher density of spines, larger dendritic diameters, and longer spines, as compared with basal dendrites (Benavides-Piccione et al., 2013). Here, we show that apical spines have longer and thinner necks than basal spines. However, spine head volumes have similar distribution in both dendritic compartments (see Fig. 8). The similarity between basal and apical head volumes is more evident in the 40-year-old individual (Fig. 5*A*), but also present in the 85-year-old individual, when including the repaired spines (groups A, C, and D; Extended Data Fig. 5-1*A–D*). The difference in neck dimension between apical and basal is also more evident in the 40-year-old (Fig. 5*B*,*C*; Extended Data Fig. 5-1*B*). The difference in neck, but not head, morphology between apical and basal spines support the argument that different mechanisms mediate the shape of the spine neck and head. Differences in spine neck dimensions could also be related to the thicker apical dendritic shaft diameter than in basal dendrites, to maintain a balance between the electrical impedance of the spine and the dendrite.

In contrast, in mice, both head and neck dimensions showed similar distributions in apical and basal compartments (Fig. 6). Because human spines are larger than mice ones, there were fewer neck deletions (groups C and D), so the separation between head and neck was more robust and this could lead to the difference in results between humans and mice. Also, in mice, there are smaller differences in diameter between apical and basal dendritic shafts, like in hippocampus (Benavides-Piccione et al., 2020, see their Figs. 5A and 9A). This is consistent with the hypothesis that spine neck dimensions compensate for dendritic shaft diameters. The more similar the dendritic shaft diameters, the more similar the spine neck thickness.

### Age-related morphologic differences in spines

In our previous work, we found that spines from basal dendrites of the younger individual (40 years old) showed more small spines, whereas in the older individual (85 years old), basal dendrites showed larger spine volumes (Benavides-Piccione et al., 2013). Despite these differences in basal dendrites, spines in apical dendrites were similar across ages, suggesting that, with aging, small basal dendritic spines are lost, or that those spines become larger. Also, the higher percentage of small spines in basal dendrites of the 40-year-old case could be related to a higher plasticity than in the older individual. Also, in the younger individual, basal dendrites had a higher proportion of short dendritic spines, whereas apical dendrites had a higher proportion of long dendritic spines. Thus, apical and basal dendrites of the younger case showed greater differences between each other. These results suggested that shorter spines of basal dendrites and longer spines of apical dendrites are lost with age.

In the present study, we show that in the older individual, there are more spines with larger head volumes and shorter necks than in the younger individual (Extended Data Fig. 5-1*G*,*H*), but the range of the distributions is similar in both individuals. This could be explained by a specific loss of “thin” spines (Dumitriu et al., 2010; Dickstein et al., 2013), or by an increase in head volumes during aging. Consistent with this, changes in the distribution of the head volumes were found in aged monkeys when analyzing “thin” and “mushroom” spines separately (Motley et al., 2018), which implies age-related increases in spine head volume.

Studying spine morphologies from human samples is important but challenging. As we analyzed age-related differences, we only had samples from two individuals of different ages. To increase the statistical effectiveness, we examined also intraindividual differences by analyzing separately different dendrites in each individual, and these results confirm our basic conclusions (Extended Data Fig. 5-2). Nevertheless, future studies of age-related differences in spine morphologies with more human samples are needed to confirm our results.

### A continuum of spine morphologies in human spines

We explored whether human spines belonged to morphologic subtypes or are part of a continuum of morphologic shapes. Statistical tests could not reject the unimodal hypothesis, meaning that we cannot prove the existence of distinct types of spines. This conclusion is of course limited to our dataset and our measured variables, so we cannot rule out the possibility that in different datasets, or with different morphologic measurements, one could identify different subtypes of spines. However, the simplest interpretation of our results is that spines represent a continuum of morphologies, without any clear subtypes. This continuous distribution of spine morphologies, which we find in human and mouse samples, is consistent with our previous studies in humans using LM (Benavides-Piccione et al., 2002, 2013) and in mice using EM (Ofer et al., 2021). A recent probabilistic analysis of spine length, width, size, and curvature of 3D reconstructed human spines revealed the existence of clusters (Luengo-Sanchez et al., 2018). However, spines could not be clearly assigned to morphologic classes because of the transitions between shapes. The unimodality of spine variables analyzed is inconsistent with previous attempts to classify spines into morphologic subtypes, such as stubby, thin, and mushroom. These categories should instead be interpreted as shorthand description of a reality where spines have a rich distribution of different morphologies.

### Correlation between head and neck morphologies

Inspection of the complete spines (group A) revealed a significant positive correlation between head volume, neck length, and neck diameter in humans and mice (Fig. 7*D–F*). When including also the repaired spines (groups A, C, and D), no correlation between head volume and neck length in mice, and a weak correlation in humans were found (Extended Data Fig. 7-1*F*). This correlation was found separately, in the apical and basal dendrites, and in the 40- and in the 85-year-old individuals (Extended Data Fig. 7-2). These differences can be explained as a result of discarding of incomplete spines (groups C and D) that tend to have longer and thinner necks. Previous studies, in human and mouse cortex, reported no correlation between head volume and neck length (Benavides-Piccione et al., 2002; Arellano et al., 2007; Ofer et al., 2021), but a correlation between head volume and neck diameter (Arellano et al., 2007; Ofer et al., 2021). This difference can be a result of longer necks that were found in the automatic 3D measurement and not in manual measurement. The higher number of analyzed spines in this study also can explain the significant correlation found here although the weak correlation.

### Functional considerations

We find that human spines have bigger heads, and longer and thicker necks than mouse spines. These differences are large (Fig. 7*A–C*), far beyond differences in spine morphologies observed between different brain regions, layers, cell types, locations along the dendritic tree, or individual ages. The morphologic differences between human and mouse spines could be significant in electrical properties or information processing capabilities (Benavides-Piccione et al., 2002, 2020; Mohan et al., 2015; Fişek and Häusser, 2020; Beaulieu-Laroche et al., 2021). One can explore this with a passive electrical model, where the spine neck electrical resistance is proportional to the neck length and inversely proportional to its radius:

$$R_{neck}\propto \frac{l}{\pi r^{2}},$$

where $R_{\text{neck}}$ is the resistance of the neck, *l* is the neck length, and *r* is the neck radius.

Using the median values of spine neck length and radius from the complete spines (group A), in humans (0.594 μm, 170 nm) and mice (0.469 μm, 134 nm), and assuming a similar cytosolic electrical resistance, we calculated the ratio between neck resistances in humans and mice of 1.27. Thus, neck resistance of mouse spines is, on average, 27% higher than that of human spines. When considering all spines (groups A, C, and D), the ratio is 1.24. Also, the ratio between apical and basal dendrites and 40- and 85-year-old individuals is 1.035 and 1.006, respectively, meaning that the spine necks in these samples should have similar resistances.

Thus, although the spine necks are longer in humans than in mouse spines, the neck electrical resistance, and thus the spine head isolation, could be lower, because of thicker neck diameters. As spine neck resistance of mice spines was recently estimated to be on average 226 MΩ (Cornejo et al., 2022), average human spine neck resistances would be around 180 MΩ, based on group A spines. A previous study of human spines estimated a lower neck resistance of 50–80 MΩ (Eyal et al., 2018), which may be related to a different population of spines (and different spine necks diameters) included within each study.

Differences in electrical isolation of spines in humans could affect the functional of computations of individual spines. A smaller electrical isolation of human spines, together with the larger spine head, which is proportional to synaptic strength (Schikorski and Stevens, 1999, 2001; Arellano et al., 2007), implies that the functional impact of human spines, and the current that they inject into the dendrites, could be larger than those from mouse neurons (Benavides-Piccione et al., 2002), resulting in larger EPSPs in the dendrite. Human membranes also have lower capacitance and different active membrane properties that affects dendritic activity, generating larger EPSPs and reduced dendritic delay (Eyal et al., 2016; Kalmbach et al., 2018). These properties could enable more reliable synapses and the transmission of higher frequency of spike trains and support the broader range of electrical frequencies that exist in the human brain (Bragin et al., 1999; Eyal et al., 2014; Testa-Silva et al., 2014; Wang et al., 2016).

## Synthesis

Reviewing Editor: Nathalie Rouach, College de France Decisions are customarily a result of the Reviewing Editor and the peer reviewers coming together and discussing their recommendations until a consensus is reached. When revisions are invited, a fact-based synthesis statement explaining their decision and outlining what is needed to prepare a revision will be listed below. The following reviewer(s) agreed to reveal their identity: Valentin Nägerl, Jörg Breustedt.

This study reports on the morphological diversity of dendritic spines in layer 3 pyramidal neurons in human and mouse cortex, imaged and reconstructed with confocal light microscopy, which offers quantitative comparative analysis of some basic morphological parameters. It is performed by two outstanding and expert labs working on the subject. This is a perennial subject in cellular and systems neuroscience as spines play such an important role for brain function. Spine properties are compared between different ages in human samples and between species, i.e. mice and human. The authors tackle the problem of faithfully recovering the spine neck dimensions in light microscopy with a computational repair approach, increasing the data yield from a given sample.

The main findings of this work are: a continuum of spine morphologies exists in both human and mice tissue samples, indicating a lack of specific spine classes such as stubby, mushroom or others.

The spine features head-volume, neck length and neck diameter are all found to be larger in human than in mice neurons. A clear positive correlation between those parameters is detected in both mice and human. Finally, the age comparison within human neurons reveals larger head volumes in the older individual (85) in both apical and basal dendritic spines and also longer apical necks.

To study the details of spine morphology in human brain tissue is important and exciting, and doing so in a comparative way with mouse tissue is very valuable. In addition, this study addresses the very relevant question of whether spines can or should be classified into specific sub-categories. The authors tested the computational method for repairing spine necks by comparing data from repaired spines to spines without the necessity for repair (figure 4-1). With this approach the validity of the process is well comprehensible. Furthemore the authors show what effect the incorporation of the computationally treated data have on the overall statistics (e.g. figure 5-1). This sophisticated analysis of the spine morphology data definitely sets standards in this area of research. Furthermore the reanalysis of the human spine data extends previously published data from the group substantially, in particular considering the importance of plasticity of the spine neck. The presented spine-repair methodology is also especially significant, although the utility/possibility to incorporate the additionally gained spine data into the overall statistics remains a little underexplained or discussed. Overall the work nicely presents and explores the advantages as well as the frontiers of spine analysis by means of confocal light microscopy, enabling the audience to better place such data into context with those gained from different imaging modalities such as STED- or electron microscopy.

Major comments:

*given that only two brains from human subjects were involved, it is really an N-of-2 case study: How much variability is there from human to human brain? The observed age differences are they real or not? The same goes for the differences with the mouse samples.

*the confocal approach clearly can’t capture the geometry of the spine neck, in view of EM and super-resolution analyses, which kind of invalidates the neck diameter analysis of the study.

*the analysis suffers from a high fraction of unconnected spines, it remains a bit unclear how the analytical “spin neck repair” procedure may bias the quantitative results.

*the paper should describe a bit more the unsupervised machine-learning algorithms that were used. Consider including a diagram to explain the analysis workflow of the study.

Minor questions or suggestions for further analysis or discussion:

- Were the imaging data deconvolved before analysis?

- Would it be possible to analyze intra-individual differences in the human spine data, for example with dendritic branch wise comparisons? This could aide to assess the age related inter-individual differences.

- Perhaps the discussion could be expanded regarding the relevance and meaning of the correlation of the spine morphology parameters the authors found here, especially in light of the differences to previously published data by the group.

- To what extent is it possible to incorporate the data from repaired spines into the overall statistics as done in Figure 5-1 and 8, considering that there is a systematic increase in the neck length compared the complete spines and no alteration in head-volume. Basically, what is the actual benefit of the procedure in terms of gaining more spine data. Perhaps this could be clarified in the discussion.

- Please add the formula for sphericity calculation to the methods as in Ofer 2021.

- Please state the units of the ordinate in the histograms (Figure 5,6), at least once in the methods, since it is not counts.

- Line 246 (p12): All morphological parameters.......the data for the spine necks do not seem to span 2-3 orders of magnitude, but spread across barely one order of magnitude. Please state more carefully. Also this makes the usefulness of a logarithmic plot on the horizontal axis for these data-sets somewhat questionable.
