# Supplementary figures and images for: Structural Analysis of Human and Mouse Dendritic Spines Reveals a Morphological Continuum and Differences across Ages and Species
Source: eNeuro. 2022 Jun 7;9(3):ENEURO.0039-22.2022. doi: 10.1523/ENEURO.0039-22.2022 (PMC9186112; doi:10.1523/ENEURO.0039-22.2022)

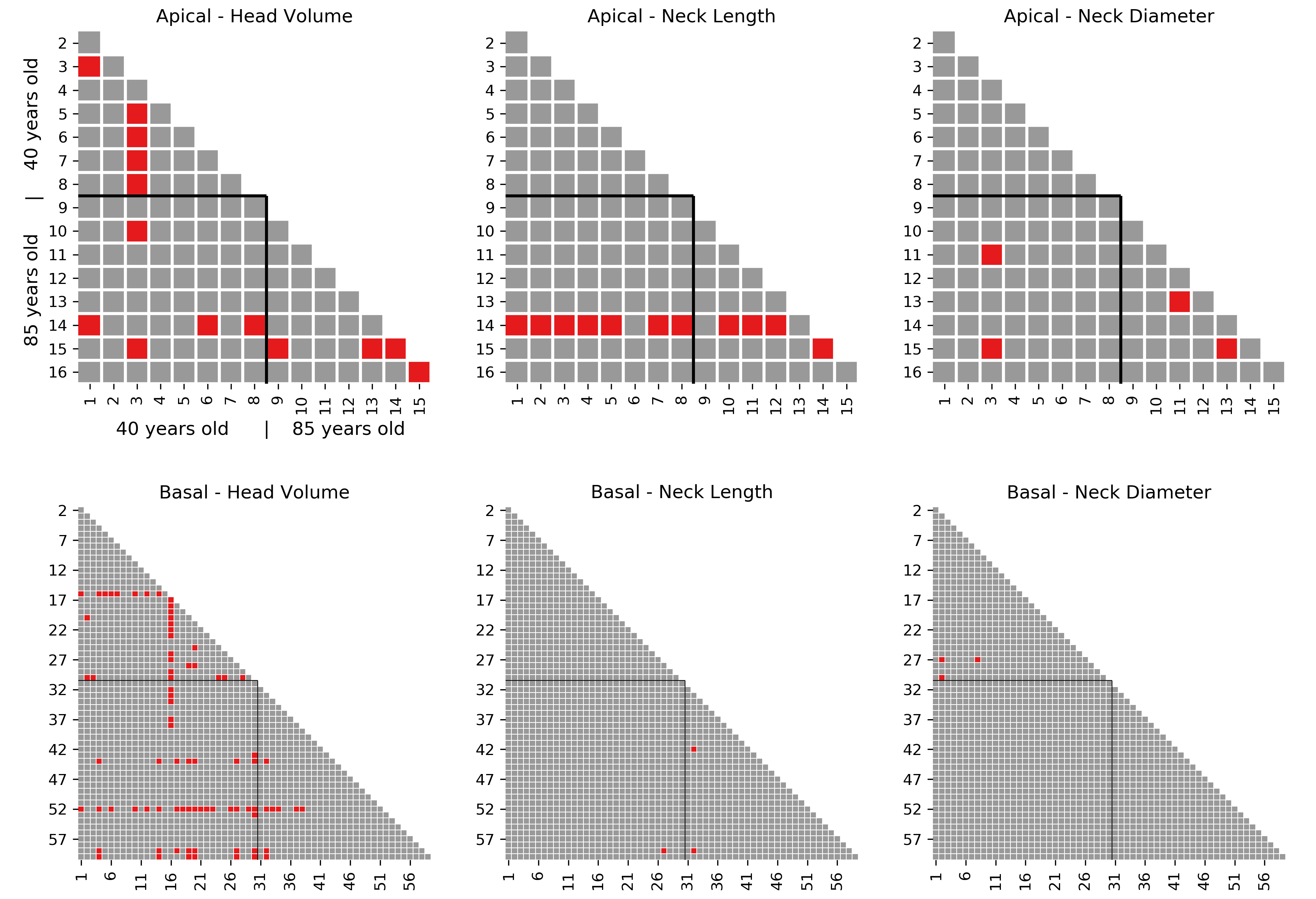

Supplement: Extended Data Figure 5-2 — Intraindividual analysis of the dendrites from the 40- and 85-year-old humans. Tukey’s pairwise multiple comparison test for the head volume, neck length, and neck diameter in apical and basal dendrites; complete spines (group A). Red squares indicate that the two varieties are significantly different (p < 0.05). Download Figure 5-2, TIF file. [file enu-eN-NWR-0039-22-s05.tif]
