# Supplementary material for: Structural Analysis of Human and Mouse Dendritic Spines Reveals a Morphological Continuum and Differences across Ages and Species
Source: eNeuro. 2022 Jun 7;9(3):ENEURO.0039-22.2022. doi: 10.1523/ENEURO.0039-22.2022 (PMC9186112; doi:10.1523/ENEURO.0039-22.2022)
Supplement: Extended Data Table 1-1 — The head volume and neck length values of the apical and basal spines from the 40- and 85-year-old individuals of the complete and repaired spines (groups A, C, and D) Download Table 1-1, DOCX file. [file enu-eN-NWR-0039-22-s09.docx]

|  | age |  | Average ± STD | median | range |
| --- | --- | --- | --- | --- | --- |
| Head volume (µm^3^) | 40 | apical | 0.3665±0.26 | 0.297 | 0.0274 - 1.7596 |
|  |  | basal | 0.348±0.23 | 0.2977 | 0.0324 - 2.3225 |
|  | 85 | apical | 0.3923±0.25 | 0.3414 | 0.0379 - 2.4035 |
|  |  | basal | 0.4087±0.26 | 0.3544 | 0.019 - 2.1746 |
| Neck length (µm) | 40 | apical | 1.0438±0.65 | 0.8836 | 0.0314 - 4.0011 |
|  |  | basal | 0.885±0.56 | 0.7381 | 0.0353 - 5.6753 |
|  | 85 | apical | 0.9732±0.64 | 0.7867 | 0.1865 - 4.158 |
|  |  | basal | 0.9038±0.57 | 0.7342 | 0.0818 - 5.1498 |

**Table 1-1.** The head volume and neck length values of the apical and basal spines from the 40 and 85 years-old individuals of the complete and repaired spines (groups A, C, and D).
