# Supplementary material for: Structural Analysis of Human and Mouse Dendritic Spines Reveals a Morphological Continuum and Differences across Ages and Species
Source: eNeuro. 2022 Jun 7;9(3):ENEURO.0039-22.2022. doi: 10.1523/ENEURO.0039-22.2022 (PMC9186112; doi:10.1523/ENEURO.0039-22.2022)
Supplement: Extended Data Table 2-1 — The head volume and neck length values of the apical (n = 153) and basal (n = 290) spines from mice of the complete and repaired spines (groups A, C, and D) Download Table 2-1, DOCX file. [file enu-eN-NWR-0039-22-s10.docx]

|  |  | Average ± STD | median | range |
| --- | --- | --- | --- | --- |
| Head volume (µm^3^) | apical | 0.176±0.13 | 0.1384 | 0.0151 - 0.9041 |
|  | basal | 0.1645±0.12 | 0.1241 | 0.0161 - 0.7097 |
| Neck length (µm) | apical | 0.7164±0.4 | 0.6382 | 0.0195 - 2.4059 |
|  | basal | 0.6938±0.38 | 0.5871 | 0.2074 - 2.2687 |

**Table 2-1.** The head volume and neck length values of the apical (n=153) and basal (n=290) spines from mice of the complete and repaired spines (groups A, C, and D).
